# *Plasmodium vivax* and *Plasmodium falciparum* at the Crossroads of Exchange among Islands in Vanuatu: Implications for Malaria Elimination Strategies

**DOI:** 10.1371/journal.pone.0119475

**Published:** 2015-03-20

**Authors:** Chim W. Chan, Naoko Sakihama, Shin-Ichiro Tachibana, Zulkarnain Md Idris, J. Koji Lum, Kazuyuki Tanabe, Akira Kaneko

**Affiliations:** 1 Island Malaria Group, Department of Microbiology, Tumor and Cell Biology, Karolinska Institutet, Stockholm, Sweden; 2 Laboratory of Malariology, Research Institute for Microbial Diseases, Osaka University, Osaka, Japan; 3 Laboratory of Evolutionary Anthropology and Health, Binghamton University, Binghamton, New York, United States of America; 4 Department of Anthropology, Binghamton University, Binghamton, New York, United States of America; 5 Department of Biological Sciences, Binghamton University, Binghamton, New York, United States of America; 6 Department of Parasitology, Graduate School of Medicine, Osaka City University, Osaka, Japan; 7 Institute of Tropical Medicine, Nagasaki University, Nagasaki, Japan; Université Pierre et Marie Curie, FRANCE

## Abstract

Understanding the transmission and movement of *Plasmodium* parasites is crucial for malaria elimination and prevention of resurgence. Located at the limit of malaria transmission in the Pacific, Vanuatu is an ideal candidate for elimination programs due to low endemicity and the isolated nature of its island setting. We analyzed the variation in the merozoite surface protein 1 (*msp1*) and the circumsporozoite protein (*csp*) of *P*. *falciparum* and *P*. *vivax* populations to examine the patterns of gene flow and population structures among seven sites on five islands in Vanuatu. Genetic diversity was in general higher in *P*. *vivax* than *P*. *falciparum* from the same site. In *P*. *vivax*, high genetic diversity was likely maintained by greater extent of gene flow among sites and among islands. Consistent with the different patterns of gene flow, the proportion of genetic variance found among islands was substantially higher in *P*. *falciparum* (28.81–31.23%) than in *P*. *vivax* (-0.53–3.99%). Our data suggest that the current island-by-island malaria elimination strategy in Vanuatu, while adequate for *P*. *falciparum* elimination, might need to be complemented with more centrally integrated measures to control *P*. *vivax* movement across islands.

## Introduction

Renewed commitment to control malaria over the last decade has resulted in major reductions in case incidence and disease mortality rates, and 32 of 99 countries with endemic malaria are pursuing an elimination strategy [1,2]. Outside of sub-Saharan Africa, *Plasmodium vivax* infections present unique and additional challenges for elimination due to the parasite’s propensity to relapse and the limitations of primaquine [1,3]. Further, malaria resurgence has the potential to undermine control and elimination efforts [4–6]. To this end, parasite population genetics studies are fundamental in identifying routes of transmission and gene flow, such that appropriate strategy for control and intervention might be implemented [7].

Islands provide an ideal model for natural ecological experiments and present a great opportunity for intervention studies. Vanuatu is an archipelago consisting of 68 inhabited islands located at the southeastern limit of malaria transmission in the Pacific. Malaria is mainly hypo- to meso-endemic, with a general decrease in annual parasite incidence (API) from the northwest to the southeast. *P*. *falciparum* and *P*. *vivax* are the predominant species, with a slightly higher prevalence of the latter especially on the southern islands [8]. Since the early 1990s, transmission rates have decreased as a result of malaria control measures and general improvement in health of the community [9,10]. On the southernmost island of Aneityum, a comprehensive elimination program was initiated in 1991 and elimination was achieved with a high degree of commitment from the local community in 1999 [11]. The Aneityum Project served as a proof of principle for the intensification of the malaria control program with the ultimate goal of elimination [9,10].

Previous population genetics studies of *P*. *falciparum* and the malaria vector *Anopheles farauti s*.*s*. in Vanuatu showed that populations were largely isolated on individual islands, with little gene flow among islands [12,13]. These findings implied that malaria control measures might be carried out on an island-by-island basis, which is the strategy currently used in the Pacific [10].

Merozoite surface protein 1 (MSP1) and circumsporozoite protein (CSP) are major surface antigens in *P*. *falciparum* and *P vivax*. These antigens are highly polymorphic, making them useful markers for assessment of parasite genetic diversity [7]. Earlier we examined *msp1* and *csp* polymorphisms in parasites from Vanuatu in the context of vaccine development for *P*. *falciparum* [14] and persisting humoral immunity after elimination on Aneityum Island for *P*. *vivax* [5]. In this study, using *msp1* and *csp* data previously generated for other aspects of malaria control, we compared the patterns of gene flow and population genetic structures in *P*. *falciparum* and *P*. *vivax* from seven sites on five islands in Vanuatu, and discussed the implications of our results in relation to the current malaria elimination strategy.

## Materials and Methods

### Ethics Statement

This study was approved by the Ministry of Health in Vanuatu and the Ethical Research Committee of Karolinska Institutet in Sweden. Due to the lack of a standardized writing system for the local “kastom” languages in Vanuatu, verbal informed consent was obtained from all adult participants and legal guardians in the case of minors. All pertinent information about the study, including the purpose, procedures, risks, benefits, and alternatives to participation, was provided to potential participants in both Bislama (lingua franca in Vanuatu; understood by most school-aged children and adults) by AK and the “kastom” language (understood by all participants) by local interpreters. The consent procedure was witnessed by a third party (e.g. teacher, village chief, nurse from local dispensary), who also recorded the name of each participant as he/she enrolled in the study. The Ministry of Health in Vanuatu and the Ethical Research Committee of Karolinska Institutet in Sweden approved the use of this consent procedure.

### Sample collections

**Fig 1:**
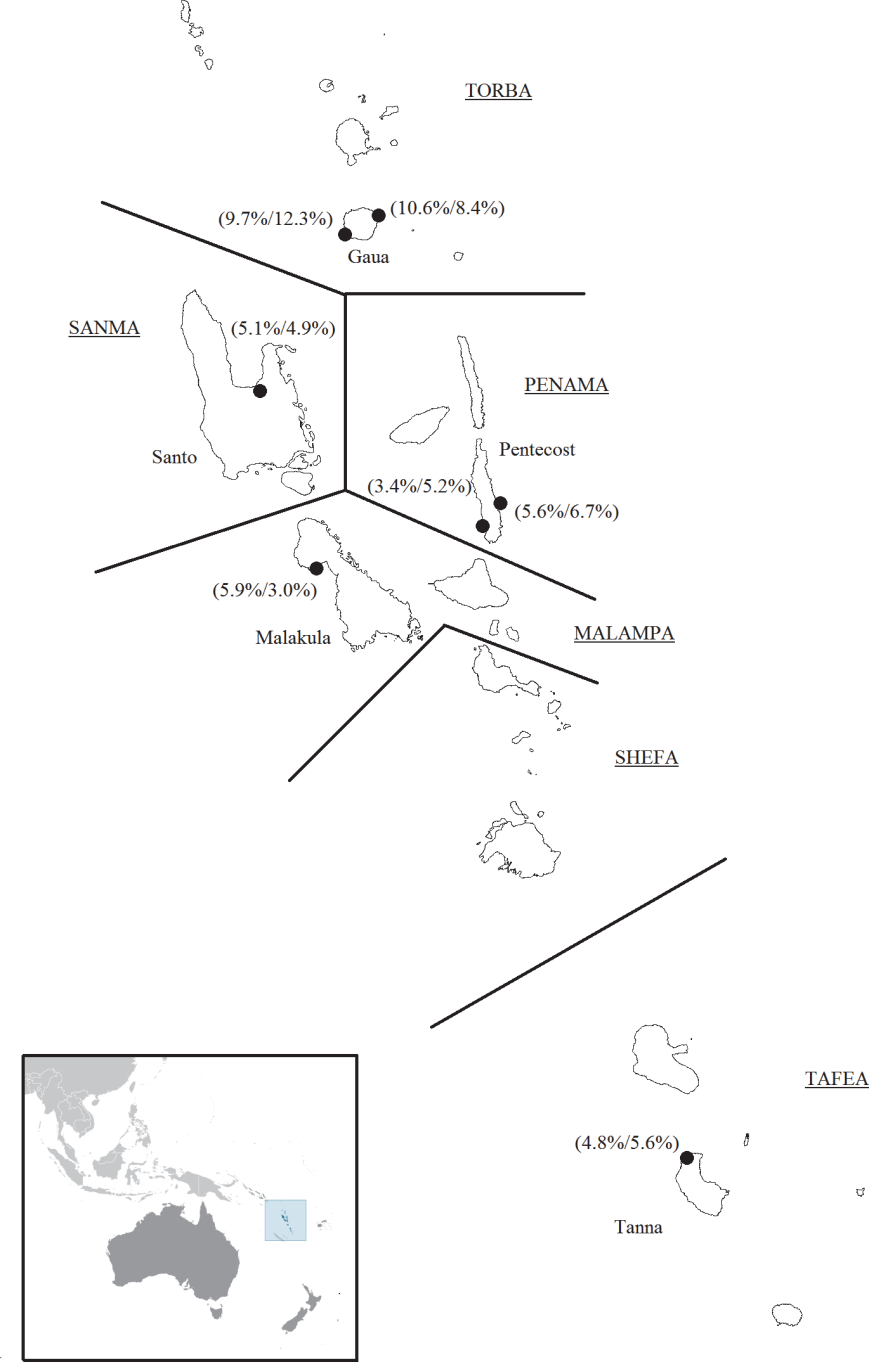
Map of Vanuatu showing the seven collection sites (black circles) on five islands. The names of the six provinces in Vanuatu are capitalized and underlined, and approximate provincial boundaries are indicated by solid lines. Species-specific parasite rates (*P*. *falciparum*/*P*. *vivax*) for each site were determined by microscopy. Maps were provided by the Library at the CIA (regional) and DIVA-GIS (Vanuatu).

*P*. *falciparum* and *P*. *vivax* isolates were collected during malariometric surveys conducted at seven sites on five islands (Gaua, Santo, Pentecost, Malakula, and Tanna) from five provinces in Vanuatu between 1996 and 2002 [5,14] (Fig. 1). Finger-pricked blood samples were collected on Whatman 31ET Chr filter paper (Whatman, Maidstone, UK) and stored desiccated [5].

### DNA extractions, PCR amplifications, and genotyping/sequences

A subset of microscopy-positive samples from each site was randomly selected for this study. Genomic DNA was extracted from blood spotted on filter paper using the QIAamp DNA Blood Mini Kit (QIAgen, Germantown, MD) according to the manufacturer’s instructions. PCR amplifications, and genotyping and/or sequencing of the merozoite surface protein 1 (*msp1*) and the circumsporozoite protein (*csp*) genes in *P*. *falciparum* [14,15] and *P*. *vivax* [5,16] were described previously. For each locus, samples with multiple alleles or genotypes were excluded for molecular analyses. The sequences described in this study have been deposited in the GenBank database (accession numbers AB116596-AB116607, AB539022-AB539045, and AB539540-AB539553).

### Molecular analyses

For each locus, unbiased haplotype diversity (*H*) for each site was calculated using the equation *H* = n(1 - ∑ X_i_^2^)/(n−1), where n is the number of haplotypes and X_i_ is the frequency of the i-th haplotype [17].

Gene flow among populations was examined at two different levels. First, gene flow was examined among the seven sites. Second, populations from the same islands were pooled and gene flow was examined among the five islands.

For each locus, pairwise F_ST_ genetic distances among sites were estimated using the program Arlequin 3.5 [18]. Genetic distances for *P*. *falciparum* and *P*. *vivax* were based on the frequencies of shared haplotypes defined by sequence polymorphisms. The statistical significance of F_ST_ distances was evaluated by randomly permuting haplotypes between sites approximately 10,000 times to generate a null distribution against which the observed value was compared. Gene flow between sites was inferred when the pairwise F_ST_ genetic distance was not statistically significant (*p* > 0.05).

Genetic variation partitioned within populations, between populations within islands, and among islands was estimated by analysis of molecular variance (AMOVA) using the program Arlequin 3.5 [18]. The statistical significance of the observed values was evaluated by randomly permuting sequences among sites approximately 1,000 times to generate a null distribution against which the observed values were compared.

## Results

### *P*. *falciparum* and *P*. *vivax* infections

Overall, PCR amplifications of *msp1* and *csp* revealed more *P*. *falciparum* infections among the seven sites in Vanuatu (Table 1). *P*. *falciparum* was the predominant species in our samples from Pentecost and Malakula (Table 1). Different PCR efficacies between the *msp1* and the *csp* amplifications were likely the cause for the slightly different numbers of infections detected in each site (Table 1). Multiple-genotype infections were more common in *P*. *vivax* than *P*. *falciparum* for both *msp1* (13.8% vs. 4.1%) and *csp* (36.2% vs. 3.6%) (Table 1).

**Table 1:** Numbers of merozoite surface protein 1 (*msp1*) and circumsporozoite protein (*csp*) sequences from seven sites in Vanuatu.

	*msp1*	*msp1*	*csp*	*csp*
Site	*P*. *falciparum*	*P*. *vivax*	*P*. *falciparum*	*P*. *vivax*
East Gaua	16 (3)	23 (1)	19 (0)	14 (8)
West Gaua	14 (0)	10 (3)	10 (0)	6 (7)
Santo	24 (2)	27 (4)	21 (0)	23 (6)
East Pentecost	25 (1)	12 (2)	26 (0)	10 (9)
West Pentecost	16 (0)	3 (2)	14 (0)	2 (4)
Malakula	62 (1)	14 (4)	61 (6)	12 (7)
Tanna	8 (0)	11 (0)	8 (0)	7 (1)
Total	165 (7)	100 (16)	159 (6)	74 (42)

The numbers of infection with multiple genotypes are given in parentheses.

### *P*. *falciparum* and *P*. *vivax* genetic diversities

Genotyping and sequencing of *msp1* and *csp* revealed that *P*. *vivax* was more genetically diverse than *P*. *falciparum* in our samples from Vanuatu. In *P*. *falciparum*, six *msp1* and five *csp* haplotypes were observed, whereas in *P*. *vivax* 14 *msp1* and 20 *csp* haplotypes were observed (Tables A-D in S1 File). All *P*. *falciparum* isolates from Tanna (n = 8) were genetically identical for both *msp1* and *csp* (Tables A and B in S1 File). In *P*. *falciparum*, *msp1* diversities ranged from 0 in Tanna to 0.692 in West Gaua, while *csp* diversities ranged from 0 in East Pentecost and Tanna to 0.733 in West Gaua (Table 2). Very few *P*. *vivax* isolates were successfully genotyped in West Pentecost (Table 1), resulting in the extreme difference in diversity estimates between the two loci (0 for *msp1* vs. 1 for *csp*; Table 2). Excluding this site, *msp1* diversities ranged from 0.697 in East Pentecost to 0.889 in West Gaua, while *csp* diversities ranged from 0.822 in East Pentecost to 0.952 in Tanna (Table 2). Haplotype diversities were significantly higher in *P*. *vivax* than *P*. *falciparum* for both *msp1* (*t*-test; *p* = 0.0135) and *csp* (*p* = 0.004) when *P*. *vivax* from West Pentecost was excluded for comparison.

**Table 2:** Haplotype diversities of *msp1* and *csp* in *P*. *falciparum* and *P*. *vivax* from seven sites in Vanuatu.

	*msp1*	*msp1*	*csp*	*csp*
Site	*P*. *falciparum*	*P*. *vivax*	*P*. *falciparum*	*P*. *vivax*
East Gaua	0.6583	0.8854	0.5906	0.8791
West Gaua	0.6923	0.8889	0.7333	0.8667
Santo	0.6703	0.8234	0.5286	0.9012
East Pentecost	0.5133	0.6970	0.0000	0.8222
West Pentecost	0.4583	0.0000	0.3626	1.0000
Malakula	0.5600	0.8791	0.7098	0.8788
Tanna	0.0000	0.7091	0.0000	0.9524

### Patterns of gene flow

#### Seven-site analyses

Analyses of F_ST_ genetic distances showed that gene flow among *P*. *falciparum* populations was restricted. Between populations, gene flow in *msp1* was limited to those from the same islands (Gaua and Pentecost; Table 3), while gene flow in *csp* was observed between the two populations on Gaua, between West Gaua and Malakula, and between Tanna and the two populations on Pentecost (Table 3).

**Table 3:** Pairwise F_ST_ genetic distances based on *msp1* (lower triangle) and *csp* (upper triangle) haplotype frequencies in *P*. *falciparum* from seven sites in Vanuatu.

Site	East Gaua	West Gaua	Santo	East Pentecost	West Pentecost	Malakula	Tanna
East Gaua		−0.001*	0.100	0.614	0.346	0.188	0.463
West Gaua	−0.065*		0.160	0.647	0.301	0.055*	0.440
Santo	0.308	0.288		0.759	0.509	0.275	0.646
East Pentecost	0.193	0.217	0.386		0.240	0.327	0.000*
West Pentecost	0.338	0.340	0.391	0.068*		0.133	0.088*
Malakula	0.106	0.142	0.324	0.067	0.251		0.251
Tanna	0.591	0.585	0.209	0.645	0.708	0.512	

Gene flow as defined by non-statistically significant (*p* > 0.05) F_ST_ distance is indicated by an asterisk (*).

In contrast, a greater degree gene flow among *P*. *vivax* populations was observed. Examination of the *msp1* F_ST_ distances revealed gene flow among all *P*. *vivax* populations in northern and central Vanuatu except those between East Gaua and West Pentecost (Table 4). The population in Tanna remained genetically distinct from all other populations, however (Table 4). For *csp*, gene flow was observed among all populations on Santo, Pentecost, Malakula, and Tanna. The two populations on Gaua were genetically distinct from those in Santo and East Pentecost. Also, East Gaua was genetically distinct from Malakula, while West Gaua was distinct from Tanna (Table 4).

**Table 4:** Pairwise F_ST_ genetic distances based on *msp1* (lower triangle) and *csp* (upper triangle) haplotype frequencies in *P*. *vivax* from seven sites in Vanuatu.

Site	East Gaua	West Gaua	Santo	East Pentecost	West Pentecost	Malakula	Tanna
East Gaua		0.034*	0.075	0.123	0.056*	0.094	0.017*
West Gaua	−0.005*		0.073	0.129	0.094*	0.089*	0.089
Santo	0.014*	0.011*		−0.004*	0.008*	0.001*	0.028*
East Pentecost	0.056*	0.041*	−0.029*		0.132*	0.045*	0.063*
West Pentecost	0.203	0.164*	0.210*	0.193*		−0.043*	-0.041*
Malakula	0.003*	−0.023*	−0.034*	−0.028*	0.166*		0.065*
Tanna	0.164	0.114	0.205	0.264	0.438	0.170	

Gene flow as defined by non-statistically significant (*p* > 0.05) F_ST_ distance is indicated by an asterisk (*).

#### Five-island analyses

**Fig 2:**
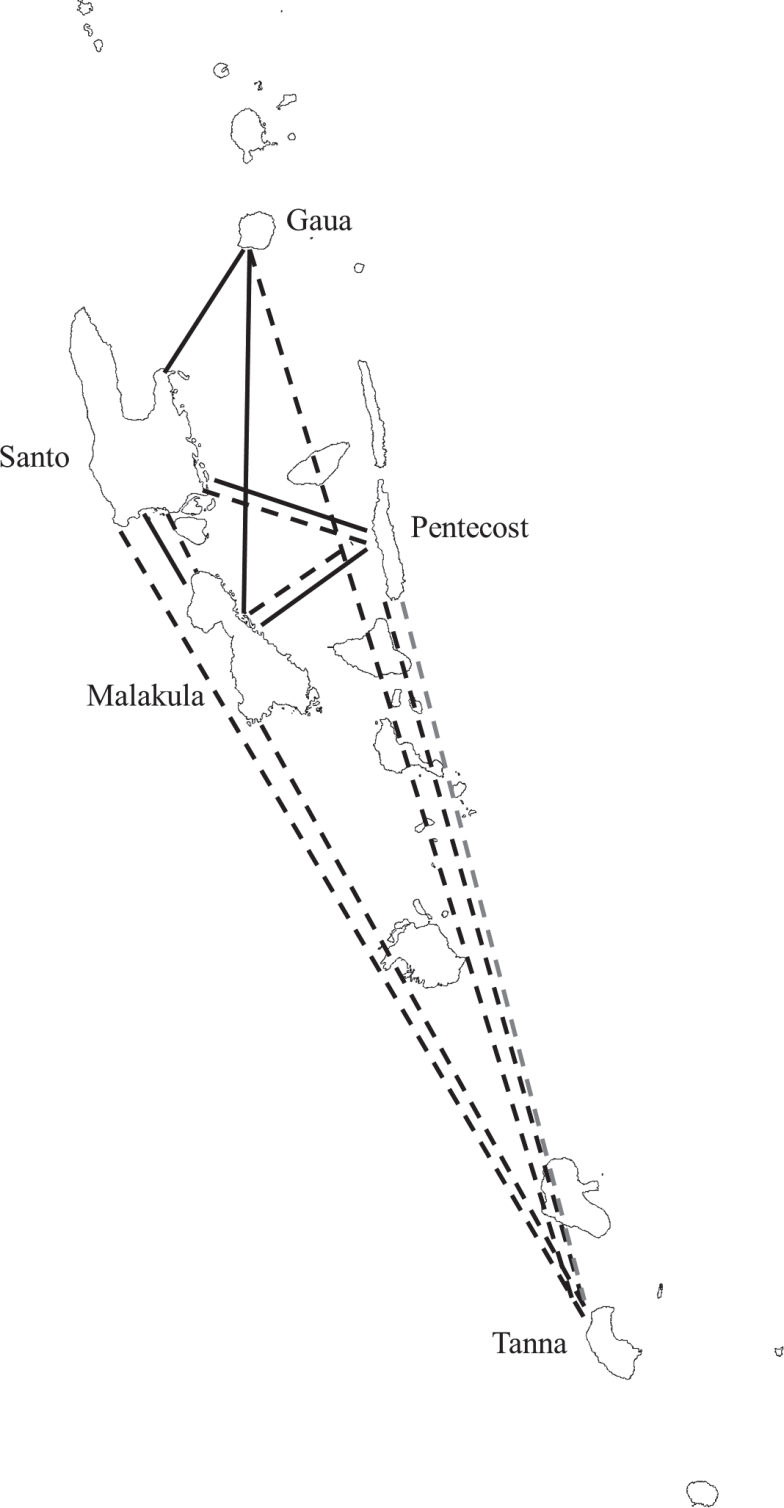
Gene flow among *P*. *falciparum* (gray) and *P*. *vivax* (black) populations from five islands in Vanuatu. Solid lines represent inferred gene flow based on merozoite surface protein 1 (*msp1*) F_ST_ genetic distances, while dotted lines represent gene flow based on circumsporozoite protein (*csp*) distances. No gene flow was observed in *P*. *falciparum msp1*. The map of Vanuatu was provided by DIVA-GIS.

Analyses of F_ST_ distances among islands revealed patterns of gene flow consistent with those from the seven-site analyses. For *P*. *falciparum*, gene flow among populations on different islands was very minimal. *P*. *falciparum* populations in central Vanuatu (Santo, Pentecost, and Malakula) were significantly differentiated from one another, despite relatively small distances separating these islands. Gene flow between Pentecost and Tanna was observed as a result of the significant sharing of the 42NE haplotype in *csp* (Fig. 2; Table B in S1 File). For *P*. *vivax*, gene flow among populations on different islands was more widespread. Gene flow among islands in central Vanuatu (Santo, Malakula, and Pentecost) was evident in both *msp1* and *csp* (Fig. 2). However, gene flow between the peripheral islands of Gaua and Tanna and the central islands was more limited. For Gaua, gene flow with Santo and Malakula was observed for *msp1* only, while for Tanna, gene flow with all other islands was observed for *csp* only (Fig. 2).

### Partitioning of genetic variation

Different population genetic structures for *P*. *falciparum* and *P*. *vivax* were revealed by AMOVA (Table 5). For *P*. *falciparum*, while most of the genetic variation (66.7–70.7%) was found within sites, variation among islands was substantial (28.8–31.2%) and statistically significant (*p* = 0.014 for *msp1* and *p* = 0.028 for *csp*). This is consistent with the F_ST_ genetic distance analyses described above that showed significant genetic differentiation among most islands (Fig. 2). In contrast, almost all genetic variation (93.1–94.8%) in *P*. *vivax* was found within sites, and the lack of significant variation among islands (*p* = 0.221 for *msp1* and *p* = 0.510 for *csp*) was in agreement with gene flow among islands as revealed in the F_ST_ genetic distance analyses (Fig. 2).

**Table 5:** Percentages of genetic variance partitioned at different population levels using analysis of molecular variance (AMOVA).

	*P*. *falciparum*	*P*. *falciparum*	*P*. *vivax*	*P*.*vivax*
Source of variation	*msp1*, %	*csp*, %	*msp1*, %	*csp*, %
Among islands	28.81*	31.23*	3.99	-0.53
Within island, between sites	0.53	2.06	2.93	5.77
Within sites	70.66	66.71	93.07	94.77

* *p* < 0.05

## Discussion

In Vanuatu, *P*. *falciparum* and *P*. *vivax* are the major malaria species, with a slight predominance of the latter [8]. In our samples we observed more *P*. *falciparum* than *P*. *vivax* infections, especially on Pentecost and Malakula (Table 1). Such difference might reflect the seasonal fluctuations in species prevalence in Vanuatu. Malaria transmission in Vanuatu is perennial. While incidence of *P*. *vivax* shows little seasonal fluctuation, incidence of *P*. *falciparum* peaks during the rainy season, from November to April [8].

Despite a slightly higher prevalence in our samples, *P*. *falciparum* showed consistently less genetic diversity than *P*. *vivax* in both *msp1* and *csp* across all sites except in West Pentecost, where few *P*. *vivax* isolates were found (Tables 1 and 2). Structural difference in orthologous genes between these two species may partially account for the difference in genetic diversity observed. For example, in *P*. *falciparum* sequence variation in *msp1* is dimorphic (either K1 or MAD 20 allelic type) and much of the variation is limited to the presence (or absence) and length of unique nine base-pair repeats in block 2 [19]. In contrast, *msp1* in *P*. *vivax* contains multiple variable blocks with extensive variation in repeats and nucleotide substitutions, and numerous potential recombination sites within and between variable blocks [20]. Direct comparison of genetic diversity in these orthologous loci between species may not be straightforward, nonetheless our result of lower genetic diversity in *P*. *falciparum* than in sympatric *P*. *vivax* was consistent with previous studies using neutral microsatellites [21,22] and other surface antigens such as apical membrane antigen 1 (*ama1*) [23,24].

Compared to *P*. *falciparum*, multiple-genotype infections were more common in *P*. *vivax* (Table 1). High frequencies of multiple-genotype infections facilitate meiotic recombination in the *Anopheles* mosquito vectors, leading to generation of novel genotypes [25] and greater genetic diversity in *P*. *vivax* (Table 2). In our *P*. *vivax* samples, the higher frequency of multiple-genotype infections in *csp* vs. *msp1* (36.2% vs. 13.8%; Table 1) was consistent with previous results from Thailand [26] and India [27].

Both MSP1 and CSP are major surface antigens, and high levels of polymorphisms in these loci are known to be a result of selection by host immunity [7,14]. However, it remains unclear whether the selective pressure on, and by extension genetic diversity in the orthologous loci of *P*. *falciparum* and *P*. *vivax* are directly comparable [7]. Differential *msp1* and *csp* genetic diversity in our samples might reflect differential selection by host immunity, i.e. stronger immune selection on the *P*. *falciparum* orthologs reduced genetic diversity observed in our samples. Moreover, specific host immune response to MSP1 and CSP may differ between *P*. *falciparum* and *P*. *vivax*, resulting in different patterns of selection seen among the orthologs [28,29]. It has been shown that in *P*. *vivax*, rapid expansion and contraction of repeats in *csp* by slipped-strand mispairing was driven by immune selection [29], consistent with our observation of related and near-identical *csp* haployptes (e.g. VC06/13/16/19; Table D in S1 File) and high frequencies of *csp* multiple-genotype infections (Table 1).

At the global level different evolutionary histories of *P*. *falciparum* and *P*. *vivax* likely contributed to the high level of genetic diversity seen in the latter [30], however in Vanuatu the role of population history in shaping parasite genetic diversity is not well understood. *P*. *vivax* is believed to have accompanied *Homo sapiens* when the latter first settled the Pacific > 40,000 years before present (ybp), compared to the relatively recent arrival of *P*. *falciparum* within the last 10,000 years [31–33]. However, northern Vanuatu was first settled only 3200 ybp by Lapita migrants from the Solomon Islands [34], suggesting that both *P*. *falciparum* and *P*. *vivax* were introduced to Vanuatu at the same time [8,31,32]. Despite similar time depth within Vanuatu, the founder effect associated with the initial colonization might have been different between the two parasite species. Previous studies on *P*. *falciparum* and *P*. *vivax* population genetics showed a decrease in *P*. *falciparum* microsatellite genetic diversity in Temotu Province of the Solomon Islands when compared to Papua New Guinea, but no decrease in *P*. *vivax* [21,35], suggesting that the effective (reproductive) population sizes of the founding populations in the Solomon Islands might have been different between these two parasite species. To the southeast of the Solomon Islands, the initial introduction of malaria parasites to Vanuatu represents yet another founding event. Our observation of lower genetic diversity in *P*. *falciparum* from Vanuatu is consistent with the results from Temotu Province in the Solomon Islands [21], which was also first settled by Lapita migrants about 3200 ybp [36], further supporting the idea that genetic drift (founder effect) might have played a greater role in shaping the genetic diversity of *P*. *falciparum* than that of *P*. *vivax* in Vanuatu.

In Vanuatu, inter-island gene flow likely contributed to the higher genetic diversity in *P*. *vivax* populations in two ways. First, gene flow mitigates the loss of haplotypes due to genetic drift in isolated island populations. In *P*. *vivax*, the most abundant *msp1* and *csp* haplotypes were shared among all five sampled islands, whereas in *P*. *falciparum* no *msp1* and *csp* haplotypes were shared by more than three and four islands, respectively (Tables A and B in S1 File). Second, maintenance of distinct haplotypes in a population allows for generation of novel haplotypes by recombination. For example, in *P*. *vivax msp1* haplotypes VM03 and VM08 might have arisen from a recombination event between haplotypes VM01 and VM06 on Malakula, where all four lineages were found (Table C in S1 File). Recombination within the poly-Q sequence in block 6 of *msp1* might have further enhanced the polymorphic nature of the gene in *P*. *vivax* (Table C in S1 File). In contrast, limited recombination events [19] as a result of isolation shown here and previously [12] might have contributed to the relatively lower level of genetic diversity observed in *P*. *falciparum*.

Gene flow in *P*. *vivax* might be facilitated by its ability to form dormant hypnozoites in the host liver and the rapid development and emergence of gametocytes. Anti-hypnozoite treatment with primaquine is not usually administered to local *P*. *vivax* cases in Vanuatu [5]. Furthermore, unlike those with blood-stage parasites, *P*. *vivax* hypnozoite-carriers are asymptomatic and might therefore be less averse to long-distance travel (e.g. between islands). Once activated, latent hypnozoites develop into merozoites, which invade red blood cells to start the erythrocytic cycle of infection. In contrast to *P*. *falciparum*, *P*. *vivax* gametocytes are known to develop early, often before symptoms appear and treatments are sought, making *P*. *vivax* transmission efficient and persistent [37,38]. The period of extrinsic development of *P*. *vivax* is known to be shorter than that of *P*. *falciparum* [39], which may further facilitate *P*. *vivax* transmission. In Vanuatu, *An*. *farauti s*.*s*. is the sole malaria vector [13]. It is unknown whether the efficiency with which this vector transmits parasites is different between *P*. *falciparum* and *P*. *vivax*, and how this difference, if it exists, might affect gene flow and genetic diversity.

Even though *P*. *vivax* showed a greater degree of gene flow, the extent of parasite movement appears to be distance dependent. Gene flow among *P*. *vivax* populations from the central islands of Santo, Malakula, and Pentecost was observed for both *msp1* and *csp*, while populations from the peripheral islands of Gaua and Tanna were more isolated, showing gene flow with these central island populations in only one locus. Parasite movement among Santo, Malakula, Pentecost, and to a less extent Gaua, is consistent with the existence of traditional exchange networks in northern and central Vanuatu, where both cultural (e.g. shell, pottery, mats) and biological (e.g. kava, yams, pigs) items are transported and traded across many islands [40]. It is reasonable to hypothesize that *P*. *vivax* is also transported and exchanged among these islands, albeit unintentionally. Tanna is not known to be a part of the aforementioned traditional exchange networks, instead parasite movement and gene flow between Tanna and these other islands might reflect the recent convenience of interisland air travel [9].

As samples used in this study were collected over a span of six years (1996 to 2002), potential temporal variation in parasite populations should be considered in the interpretation of parasite genetic diversity and gene flow. We evaluated the temporal “stability” of parasite populations from four sites (Santo, Malakula, East and West Pentecost) in which there were samples from at least two years. Analyses of *msp1* and *csp* F_ST_ genetic distances revealed no year-to-year differentiation among *P*. *vivax* populations from the same site, indicating that *P*. *vivax* populations remained relatively stable over the sampled period. For *P*. *falciparum*, genetic differentiation was observed among temporal populations from Malakula (both *msp1* and *csp*) and East Pentecost (*msp1* only). Given that *P*. *falciparum* incidence in Vanuatu shows strong seasonality [8], drastic year-to-year changes in the genetic makeup of *P*. *falciparum* populations due to genetic drift during the dry season are not unexpected. More comprehensive sampling of contemporaneous parasite populations from different islands will allow for a more refined description of gene flow in both *P*. *falciparum* and *P*. *vivax*.

Distinct parasite population structures and patterns of gene flow between *P*. *falciparum* and *P*. *vivax* have important implications on the current malaria initiatives in Vanuatu. Our previous analyses of *P*. *falciparum* and *An*. *farauti s*.*s*. genetic diversities showed that these two species were largely localized to individual islands [12,13]. However for *P*. *vivax*, we demonstrated that parasite movement among islands and across provincial boundaries is common, suggesting that the current island-by-island elimination strategy might need to be complemented with more integrated control and coordination among islands and provinces [10]. Moreover, the risk of resurgence or reintroduction of parasites from other islands after elimination should not be underestimated, as shown by our own experience on Aneityum Island, where *P*. *vivax* from Tanna was responsible for the outbreak six years after initial elimination [5].

## Conclusions

In Vanuatu, *P*. *falciparum* and *P*. *vivax* were both present but showed different levels of genetic diversity and different patterns of gene flow and population structures. The high level of diversity in *P*. *vivax* populations was maintained by greater degree of gene flow among islands, which also resulted in greater genetic similarity among populations on different islands. Our data suggested that the current malaria control strategy might need to be bolstered with centrally integrated components and coordination among islands and provinces to ensure elimination and sustainable malaria freedom.

## Supporting Information

S1 File Distributions of merozoite surface surface protein 1 (msp1) and circumsporozoite protein (csp) haplotypes in *Plasmodium falciparum* and *Plasmodium vivax* from seven sites in Vanuatu. (XLSX)
